# Chronological Changes in Gonadotropin-Releasing Hormone 1, Gonadotropins, and Sex Steroid Hormones along the Brain–Pituitary–Gonadal Axis during Gonadal Sex Differentiation and Development in the Longtooth Grouper, *Epinephelus bruneus*

**DOI:** 10.3390/cells12222634

**Published:** 2023-11-16

**Authors:** Wengang Xu, Hisashi Chuda, Kiyoshi Soyano, Jun Zeng, Weiping Mei, Huafeng Zou

**Affiliations:** 1 School of Ocean, Yantai University, Yantai 264005, China; 2 Aquaculture Research Institute, Kindai University, Wakayama 649-2211, Japan; chuda@kindai.ac.jp; 3 Institute for East China Sea Research, Organization for Marine Science and Technology, Nagasaki University, Nagasaki 851-2213, Japan; soyano@nagasaki-u.ac.jp; 4 Guangxi Academy of Sciences, Nanning 530007, China; junzeng@gxas.cn (J.Z.); mei@gxas.cn (W.M.); 5 Institute of Beibu Gulf Marine Industry, Fangchenggang 538000, China; 6 Key Laboratory of Exploration and Utilization of Aquatic Genetic Resources, Ministry of Education, Shanghai Ocean University, Shanghai 201306, China; hfzou@shou.edu.cn

**Keywords:** hermaphrodite, ovary, oogonia, primary oocyte, FSH, LH

## Abstract

(1) Fshβ and Lhβ showed stronger signals and higher transcript levels from 590 to 1050 dph than at earlier stages, implying their active involvement during primary oocyte development. (2) Fshβ and Lhβ at lower levels were detected during the phases of ovarian differentiation and oogonial proliferation. (3) E_2_ concentrations increased significantly at 174, 333, and 1435 dph, while T concentrations exhibited significant increases at 174 and 333 dph. These findings suggest potential correlations between serum E_2_ concentrations and the phases of oogonial proliferation and pre-vitellogenesis.

## 1. Introduction

The longtooth grouper, *Epinephelus bruneus*, is widely distributed throughout East Asia and is highly regarded in the marine fish industry for its superior quality and commercial value [1]. This species exhibits protogynous hermaphroditism, where its gonads initially differentiate into those of a phenotypic female and later transform into those of a functional male after 7 or 9 years. In captivity, *E. bruneus* typically reaches maturity within 4 years [2]. However, the spontaneous onset of maturity and spawning remains a significant limitation in seed production. To address this challenge, exogenous hormones like human chorionic gonadotropin (hCG) are administered when parent fish reach 6–10 kg and are 5–8 years old during the spawning season to induce maturation for seed production [3]. Recent research efforts toward artificial spawning have led to an increased larval survival rate of 49–54%, and substantial work has been dedicated to advancing techniques for broodstock management, artificial fertilisation, and larval rearing [3]. Nevertheless, there is an urgent need for further technological improvements, especially in artificial propagation and seedling production for this species. Therefore, understanding the endocrine mechanisms governing early gonadal development in longtooth groupers is crucial for future species management.

Gonadal sex differentiation, development, and maturation constitute pivotal physiological processes regulated by the endocrine system, primarily the brain–pituitary–gonadal (BPG) axis, in many fish species, including groupers [4,5]. Gonadotropin-releasing hormone (GnRH), secreted by the brain, stimulates the synthesis of two types of gonadotropins (GtHs) in the pituitary gland: follicle-stimulating hormone (Fshβ) and luteinising hormone (Lhβ). Fshβ and Lhβ are responsible for triggering steroidogenesis and gametogenesis in the gonads [6]. Sex steroid hormones, 17β-ostradiol (E_2_), and testosterone (T) exert both positive and negative feedback loops to regulate the brain and play a pivotal role in the neuroendocrine regulation of reproductive behaviour in fish [7].

Previous studies have identified the presence of Fshβ and Lhβ during various stages of the teleost life cycle. For example, immune signals of Fshβ and Lhβ were detected in the pejerrey, *Odontesthes bonariensis* [8], and the gene expression of *fshβ* and *lhβ* increased during sex differentiation in the red-spotted grouper, *E. akaara* [9]. In addition, single Fshβ or Lhβ signals have been detected during primary oocyte development in species like the gilthead seabream, *Sparus aurata* [10], and the ricefield eel, *Monopterus albus* [11]. Fshβ is known to act as an active hormone in early vitellogenesis and spermatogenesis, from the onset of puberty to maturation, while Lhβ plays pivotal roles in final maturation, ovulation, or spermiation in various marine species such as the salmonid fish [12,13,14], Japanese conger, *Conger myriaster* [15]; Japanese eel, *Anguilla japonica* [16]; and European sea bass, *Dicentrarchus labrax* [17]. However, there is still a dearth of research regarding changes in the contents of the Fshβ and Lhβ during gonadal sex differentiation and development, as well as those of the steroid hormones E_2_ and T in the BPG axis of the longtooth grouper. Consequently, technological advancements in the realms of artificial reproduction and seedling production for the longtooth grouper have been impeded. Therefore, in this study, we aimed to ascertain (1) the appearance and localisation of Fshβ- and Lhβ-producing cells in the pituitary gland, (2) the gene expression of *gnrh1*, *fshβ*, and *lhβ* in the brain and pituitary gland, and (3) E_2_ and T levels in the serum from 53 to 1435 days post-hatching (dph) in the longtooth grouper.

## 2. Materials and Methods

### 2.1. Study Species

**Figure 1:**
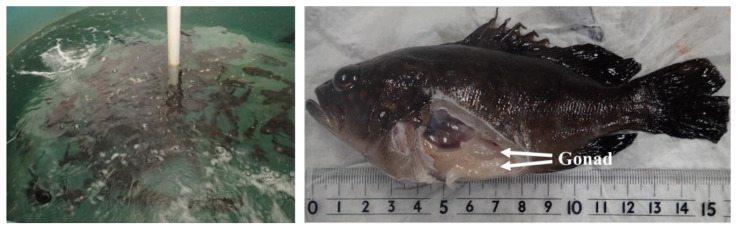
The indoor recirculating aquaculture tank and longtooth grouper, *Epinephelus bruneus*, used in this study.

The experiment was conducted at the Aquaculture Research Institute, Kindai University, Japan. Specimens of newly hatched *E. bruneus* were collected on 15 June 2015 and reared in indoor recirculating aquaculture tanks with slow-flowing and aerated sea water. The fish were exposed to a natural water temperature ranging from 2.18 ± 0.21 °C to 27.46 ± 0.37 °C and natural photoperiod conditions (Figure 1). Specimens were collected on the following dates: 7 August 2015, 7 December 2015, 13 May 2016, 2 February 2017, 9 May 2017, 12 May 2018, and 30 May 2019. The fish were fed twice daily with a commercial diet as follows: from 0 to 200 days post-hatching (dph), they were given Otohime EP2 (containing 48% crude protein and 14% crude fat), provided by Marubeni Nisshin Feed Co., Ltd., Tokyo, Japan; from 200 to 500 dph, they were fed Otohime EP4 (containing 48% crude protein and 14% crude fat); and from 500 to 1435 dph, their diet consisted of Otohime EP6 (with 51% crude protein and 9% crude fat). The mortality rate remained below 10% throughout the experimental period.

### 2.2. Sampling Procedures

Specimens from each age group were anaesthetised using 2-phenoxyethanol (Wako Pure Chemical Industries, Ltd., Osaka, Japan), and the dosages of anaesthesia for various teleosts [18] were used as base information, and the concentration was 400 mg/L. Their total length (TL), body weight (BW), and gonad weight (GW) were measured (Figure 1, Table 1). The gonadosomatic index (GSI) was calculated as follows:

GSI = GW/BW × 100%

Head tissues were removed from specimens (*n* = 20) via decapitation. However, obtaining both a single gonad and pituitary gland from fish at 53 dph proved challenging due to their small size. Consequently, for these younger specimens, the posterior section of the visceral cavity and the heads of 10 individuals were fixed in Bouin’s solution. The heads of the remaining 10 specimens were fixed using RNAlater reagent (Ambion Inc., Invitrogen Life Technologies, Waltham, MA, USA). The gonads of fish ranging from 174 to 1435 dph (*n* = 15) and brains with pituitary glands (*n* = 6) were fixed in Bouin’s solution for histological analysis. The pituitary glands and brains from another nine specimens were extracted and either fixed in RNAlater reagent or stored at −80 °C. Unfortunately, obtaining blood samples from fish at 53 dph was not feasible due to their small size. Blood from fish between 174 and 1435 dph (*n* = 15) was collected from the caudal vasculature using a heparinised syringe. Following centrifugation at 1500× *g* for 15 min, serum samples were collected and stored at −80 °C. All tissues preserved in Bouin’s solution were initially fixed for 24 h and subsequently stored in 70% ethanol at 4 °C.

### 2.3. Histological Procedures of Gonads and Pituitary Glands and Immunohistochemistry (ICH)

The histological procedures for both the gonads and IHC of entire brains with pituitary glands were performed as previously described by Xu et al. (2020, 2022) [19,20]. In brief, the gonads and entire brains with pituitary glands were embedded in paraffin, and then, cross-sectioned (thickness, 5–8 μm). The gonad sections were dehydrated using gradient ethanol and stained with haematoxylin–eosin (HE). The stages of gonadal development were determined according to the criteria set forth by Sao et al. in 2012 [21] and categorized into undifferentiated, oogonial proliferation, and primary growth oocyte phases.

Concurrently, the sections of the entire brains with pituitary glands were dehydrated and subsequently immersed in a 10 mM citric acid solution (Wako Pure Chemical Industries, Ltd., Osaka, Japan) and maintained at 90 °C for 15 min. The primary antisera used, namely, anti-mummichog (*Fundulus heteroclitus*) FSHβ or LHβ, were generously provided by Dr. Shimizu [22] and were diluted to ratios of 1:1000 and 1:5000, respectively. IHC analysis was conducted using the avidin–biotin peroxidase method with a Histofine SAB-PO (R) kit (Nichirei Biosciences Inc., Tokyo, Japan) and a Histofine DAB Peroxidase Substrate kit (Nichirei Biosciences Inc.). After the IHC experiment, these sections were lightly counterstained with haematoxylin, mounted, examined using a microscope, and photographed digitally (Olympus FX380, Olympus Corporation, Tokyo, Japan). In this study, we present the results using the best pictures chosen from the sections of six brains with pituitary glands.

### 2.4. Total RNA Extraction and cDNA Synthesis

For gene detection, total RNA was extracted from the head and the brain or pituitary gland of fish at 53 dph and 174 to 1435 dph, respectively. RNA was extracted using TRIzol^®^ reagent (Life Technologies Corp., Waltham, MA, USA). The frozen samples were left to defrost in a 2 mL tube at 4 °C for 5 min. An amount of 500 µL of TRIzol^®^ reagent was added to the tube, and the tissues were crushed using a homogeniser. Subsequently, 500 µL TRIzol^®^ Reagent was added and the mixture was centrifuged at 12,000× *g* and 4 °C for 10 min. The supernatant was transferred to a new 1.5 mL tube. Thereafter, 200 µL of chloroform (Wako Pure Chemical Industries, Ltd., Osaka, Japan) was added to the tube, stirred for 5 min, and centrifuged at 12,000× *g* and 4 °C for 15 min. After centrifugation, 500 µL of isopropanol was added to the supernatant. In addition, 3 µL of ethachinmate and 9.9 µL of sodium acetate (Nippon gene Co., Ltd., Tokyo, Japan) were added, and the mixture was incubated at 4 °C for 10 min. Subsequently, the mixture was centrifuged at 12,000× *g* and 4 °C for 10 min. An amount of 1 mL of 75% ethanol was added to the resulting supernatant. The mixture was centrifuged at 12,000× *g* and 4 °C for 5 min, and 30 µL of diethylpyrocarbonate-treated (DEPC) water was added and stirred until the RNA was dissolved. RNA quantification was conducted using a NanoDrop 2000 spectrophotometer (Thermo Scientific Inc., Waltham, MA, USA). RNA integrity was assessed using gel electrophoresis.

In a PCR tube, 1 μg of RNA from the head and brain, or 150 ng from the pituitary gland, was diluted to 10 μL with PCR-grade water. This mixture was reacted with 1 µL of anchored-oligo(dT)_18_ primer (50 pmol/µL), 2 µL of random hexamer primer (600 pmol/µL), 4 μL of transcriptase reaction buffer (5×), 0.5 µL of RNase inhibitor (40 U/µL), 2 µL of dNTPs (10 mM each), and 0.5 µL of reverse transcriptase (20 U/μL) using a Transcriptor first-strand cDNA synthesis kit (Roche Diagnostic GmbH, Mannheim, Germany). The tube, containing 20 µL of the mixture, was placed in a Gene Amp PCR System 2400 thermal cycler (Perkin-Elmer Corp., Norwalk, CT, USA) and reacted as follows: 25 °C for 10 min, 50 °C for 1 h and 85 °C for 5 min. Complementary DNA was used as a template for PCR amplification.

### 2.5. Procedures for Making Standard Plasmids

#### 2.5.1. Electrophoresis with 1.5% Agarose Gel

PCR amplification products were subjected to electrophoresis using a 1.5% agarose gel. Initially, 0.75 g of Agarose S (Nippon Gene Co., Ltd., Tokyo, Japan) was added to 50 mL of TBE buffer (1×) within a 100 mL beaker. This mixture was briefly heated in a microwave oven for 30 to 60 s. The resulting melted agarose mixture was then carefully poured into a Gel Maker Set (Cosmo Bio Co., Ltd., Tokyo, Japan), following which the comb was inserted and left for 1 h for solidification of the gel. The gel was removed and placed in a mini gel tank (Cosmo Bio Co., Ltd., Tokyo, Japan). TBE buffer (1×) was poured into the tank, and the gel was submerged within it. For the cDNA amplification, 10 µL reaction mixtures were prepared, each containing 1 µL of cDNA, 1 µL of forward and reverse primers for each gene, 1 µL of dNTPs (2.5 mM), 1 µL of buffer (10×), 5 µL of PCR-grade water, and 0.05 µL of Advantage 2 polymerase (Clontech Advantage 2 PCR kit, Clontech, Palo Alto, CA, USA). Additionally, 5 µL of Gene Ladder 100 (Nippon Gene Co., Ltd., Tokyo, Japan) was added to serve as a size reference. Electrophoresis was conducted at 100 V for a duration of 40 min. The gel was then immersed in a 500 µg/mL solution of EtBr and gently shaken on an oscillating machine (Taitec Co., Ltd., Saitama, Japan) for 30 min. Subsequently, the gel was briefly rinsed via immersion in distilled water for 10 s, and then, placed in a gel projector (ATTO Co., Ltd., Tokyo, Japan) to assess the desired products. The bands of gel containing the products of interest were cut, weighed, and stored in a 1.5 mL tube.

#### 2.5.2. Gel Extraction

The gel was extracted using a QIAquick Gel Extraction Kit (Qiagen, Hilden, Germany). The target products were cut from the gel and weighed in the tube, and they were placed on ice for 30 min. Buffer QG with a quantity of approximately three times the gel weight was added to the tube and incubated at 50 °C for 10 min. Isopropyl alcohol (Wako Pure Chemical Industries, Ltd., Osaka, Japan) in the same quantity as the gel weight was added. This mixture was then transferred into a new 2 mL collection tube and centrifuged at 12,000× *g* and 4 °C for 1 min. The substrate liquid was disposed of. An amount of 500 µL of new QG buffer was added to the spin column and centrifuged at 12,000× *g* and 4 °C for 1 min. Thereafter, the substrate liquid was discarded, and 750 µL of PE buffer was added and incubated at 4 °C for 5 min, and centrifuged at 12,000× *g* and 4 °C for 1 min. The substrate liquid in the collection tube was discarded. To remove the residual liquid in the spin column, this collection tube was centrifuged at 12,000× *g* and 4 °C for 1 min. The spin column was then placed on a new 1.5 mL tube. An amount of 50 µL of PCR-grade water was added to the spin column and incubated at 4 °C for 1 min, and then, centrifuged at 12,000× *g* and 4 °C for 1 min. The DNA concentration was measured using a NanoDrop 2000.

#### 2.5.3. Ligation and Transformation

A TOPO TA Cloning^®^ Kit (Invitrogen) and Competent Quick DH5α (Toyobo, Osaka, Japan) were used for ligation and transformation. The product of gel extraction was placed on ice for thawing. Thereafter, 2 µL of product, 0.5 µL of salt solution, and 0.5 µL of TOPO vector were added to a 1.5 mL tube, mixed via pipetting, and incubated at 4 °C for 30 min. Competent Quick DH5α was placed on ice for thawing. Thereafter, 3 µL of competent cells was added to the tube and gently shaken up and down for mixing. The mixture was then incubated on ice for 5 min. The tube was placed in a water bath (Taitec Co., Ltd., Saitama, Japan) and subjected to heat-shock treatment for 30 s at 42 °C without shaking. Immediately after this, the tube was placed on ice. In a Bechtop, 500 µL of Super Optimal broth with Catabolite repressor (SOC) medium (Invitrogen) was added to the tube. The tube was tightly capped, placed in an oscillating machine, and horizontally shaken for 1 h at 37 °C. Thereafter, 250 µL of SOC medium was added to the tube again. The tube was placed in the oscillating machine and horizontally shaken for 3 h at 37 °C. To prepare the plate, agar medium was produced with LB agar and 50 µg/mL kanamycin. This medium was poured into a plate and stored at 4 °C until further use. Once the plate was taken out, it was incubated at 37 °C for drying. Following this, 20 µL of X-gal (40 mg/mL) was spread onto the LB plate using a plastic rod. Thereafter, 50 µL of the mixture from the tube was added to the same LB plate and incubated at 37 °C overnight.

#### 2.5.4. PCR Cloning

To check whether the plasmid contained the target gene, PCR cloning was carried out using an EmeraldAmp^®^ PCR Master Mix Kit (Takara Bio Co., Ltd., Shiga, Japan); 5 µL of EmeraldAmp PCR Master Mix (2 × Premix), 1 µL of M13 forward primer (CAGGAAACAGCTATGACCATG, 10 pmol/µL), 1 µL of M13 reverse primer (GTAAAACGACGGCCAGTG, 10 pmol/µL), and 3 µL of PCR-grade water were added to the tube and mixed. The white bacterial colony was chosen and dipped using a toothpick; the colony was then immersed into the tube mixture and stirred well. The tube was then placed on a thermal cycler, and the reaction was set as follows: 40 cycles of 98 °C for 10 s, 58 °C for 30 s, 72 °C for 1 min, and finally, at 4 °C forever. After the reaction, the product was checked using electrophoresis with 1.5% agarose gel as previously described.

#### 2.5.5. Liquid Culture of Colon Bacillus

The LB medium was produced using LB broth base (Invitrogen) and ampicillin (100 µg/mL). Three millilitres of LB medium was added to a 10 mL disposable tube. The toothpick was gently dipped into the white bacterial colony, including the purpose gene, and then immersed into the LB medium and stirred well. The disposable tube was placed in the oscillating machine and horizontally shaken at 37 °C overnight. Subsequently, the bacterial culture appeared turbid, and the tube was stored at 4 °C until plasmid extraction.

#### 2.5.6. Plasmid Extraction and Sequencing Analysis

The plasmid was extracted from the *Bacillus* coli using a QIAprep^®^ Spin Miniprep Kit (Qiagen, Hilden, Germany). The bacterial culture solution was extracted and stirred well. This solution (1 mL) was then added to a 1.5 mL centrifuge tube and centrifuged at 12,000× *g* and 4 °C for 1 min. The supernatant was then discarded completely. The remaining culture solution (2 mL) was treated in the same manner. Then, 250 µL of P1 buffer, 250 µL of P2 buffer, and 350 µL of N3 buffer were added in that order, stirring well after each step. The tube was centrifuged at 12,000× *g* and 4 °C for 10 min. The spin column was then placed in a 2 mL collection tube, and the supernatant was added to the spin column and centrifuged for 1 min. After centrifugation, 750 µL of PE buffer was added to the spin column and centrifuged for 1 min. After centrifugation, the residual liquid in the spin column was discarded; centrifugation was conducted for 1 min and the liquid in the collection tube was discarded. The spin column was inserted into a new 1.5 mL tube and 20–50 µL of PCR-grade water was added to the membrane of the spin column, and then, incubated at 4 °C for 1 min. Then, the tube was centrifuged at 12,000× *g* and 4 °C for 1 min, and the plasmid concentration was measured using a NanoDrop 2000. Subsequently, the plasmid was stored at 4 °C for sequencing analysis. 

In the tube, 600 ng of plasmid, 0.64 µL of M13 forward primer (10 pmol/µL), and moderate Ultra-Pure™ distilled water (Invitrogen) were added to a final volume of 14 mL. This plasmid mixture was sequenced by Fasmac Co., Ltd. (Kanagawa, Japan).

### 2.6. Quantitative Real-Time PCR Analysis for gnrh1, fshβ, and lhβ

The primers for *gnrh1* (GenBank Accession No. FJ380047) were designed according to the GenBank database using the Primer3Plus software available online (primer3plus.com/ accessed on 15 May 2020) and manufactured by Fasmac Co., Ltd. (Kanagawa, Japan; Table 2). The mRNA levels of *gnrh1* were determined using a FastStart Essential DNA Green Master (Roche Diagnostics GmbH, Mannheim, Germany) as previously described by Xu et al. (2020, 2022) [19,20] Supplementary Materials.

The probe primers for *fshβ* (GenBank Accession No. EF583919) and *lhβ* (GenBank Accession No. EF583920) were obtained from Ryu et al. (2013) [23] and produced by Integrated DNA Technologies (Redwood City, CA, USA; Table 2). The mRNA levels of *fshβ* and *lhβ* were detected using a FastStart Essential DNA Probes Master (Roche Diagnostics GmbH, Mannheim, Germany), which was performed as previously described by Xu et al. (2020, 2022) [19,20] Supplementary Materials.

### 2.7. Quantification of Serum E_2_ and T Concentrations

Steroids were extracted from the serum samples using diethyl ether (Wako Pure Chemical Industries, Ltd., Osaka, Japan). Diethyl ether (including steroids) was evaporated using nitrogen. The dried extracts were subsequently dissolved in enzyme immunoassay (EIA) buffer. The E_2_ and T concentrations were quantified using an Estradiol EIA kit or a Testosterone EIA kit (Cayman Chemical, Ann Arbor, MI, USA) according to the manufacturer’s instructions.

### 2.8. Statistical Analysis

All data were analysed using SPSS software (version 26.0; IBM, Armonk, NY, USA). The normality of the continuous data was tested using Shapiro–Wilk tests. All data are reported as the mean ± standard deviation (SD). Statistical differences between independent samples were assessed using one-way analysis of variance (ANOVA) with Tukey’s honest significant difference (HSD) test. The one-way ANOVA model included age as the independent variable and the *gnrh1*, *fshβ*, *lhβ*, E_2_, and T contents at each age as the dependent variables. The degrees of freedom (dfs) and *p*-values were determined. Differences were considered statistically significant at *p* < 0.05.

## 3. Results

### 3.1. Gonadal Development

**Figure 2:**
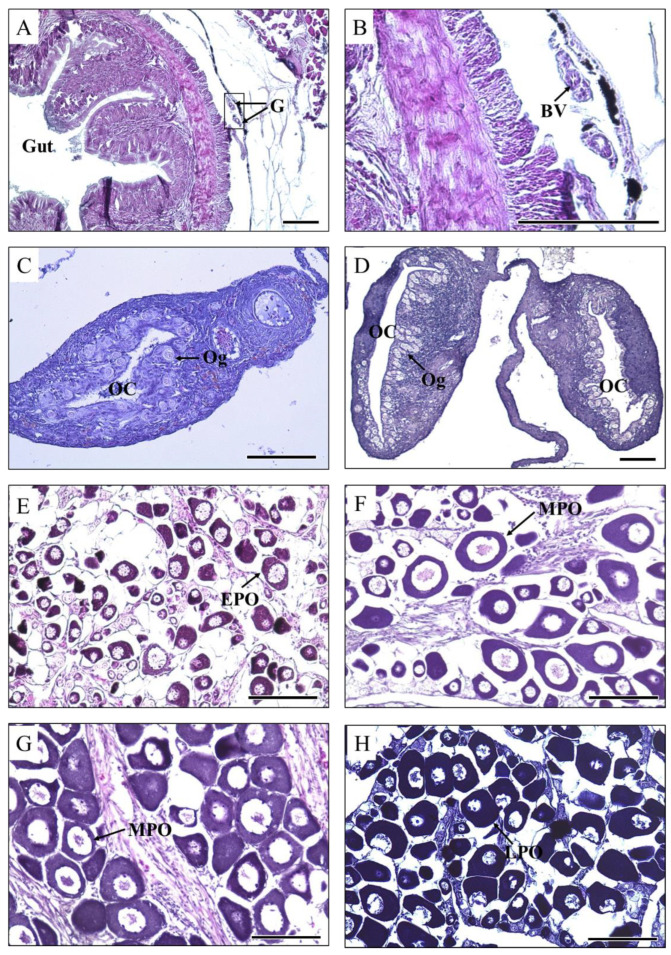
Gonadal development of the longtooth grouper, *Epinephelus bruneus*, from 53 to 1435 days post-hatching (dph). (**A**) Cross-section at 53 dph showing an undifferentiated gonad located near the gut. (**B**) High magnification of gonads marked in A. (**C**,**D**) Cross-section at 174 and 333 dph showing the fully formed ovarian cavity and oogonia. (**E**) Cross-section at 590 dph showing early-perinucleolar-stage oocytes. (**F**,**G**) Cross-section at 687 and 1050 dph showing middle-perinucleolar-stage oocytes. (**H**) Cross-section at 1435 dph showing late-perinucleolar-stage oocytes. G, gonad; BV, blood vessel; OC, ovarian cavity; Og, oogonia; EPO, early-perinucleolar-stage oocyte; MPO, middle-perinucleolar-stage oocyte; LPO, late-perinucleolar-stage oocyte. Scale bar = 100 µm.

The gonadal development of the 53 dph grouper was in the undifferentiated phase. The gonad was located near the gut (Figure 2A) and consisted of a pair of elongated lobes separated at the anterior end. Additionally, blood vessels were observed (Figure 2B). Gonadal development occurred at 174 and 333 dph during oogonial proliferation. The ovarian cavity was fully formed with a small interspace at 174 dph, and oogonia appeared around the ovarian cavity (Figure 2C,D). Gonadal development occurred during the primary growth of oocytes from 590 to 1435 dph (Figure 2E–H). At 590 dph, a few early-perinucleolar-stage oocytes with a strong basophilic cytoplasm appeared. The nuclei grew larger, and a few larger nucleoli were observed on the inner nuclear membrane. The oocyte cytoplasm volume increased and was stained with haematoxylin (Figure 2E). At 687 and 1050 dph, a few middle-perinucleolar-stage oocytes were observed, and the oocyte diameter increased. The oocyte cytoplasmic volume increased significantly (Figure 2F,G). Gonadal development at 1435 dph occurred in late-perinucleolar-stage oocytes, and the cytoplasm was stained darker with haematoxylin (Figure 2H).

### 3.2. General Pituitary Structures and Fshβ and Lhβ Immunoreactivity in the Pituitary Gland

**Figure 3:**
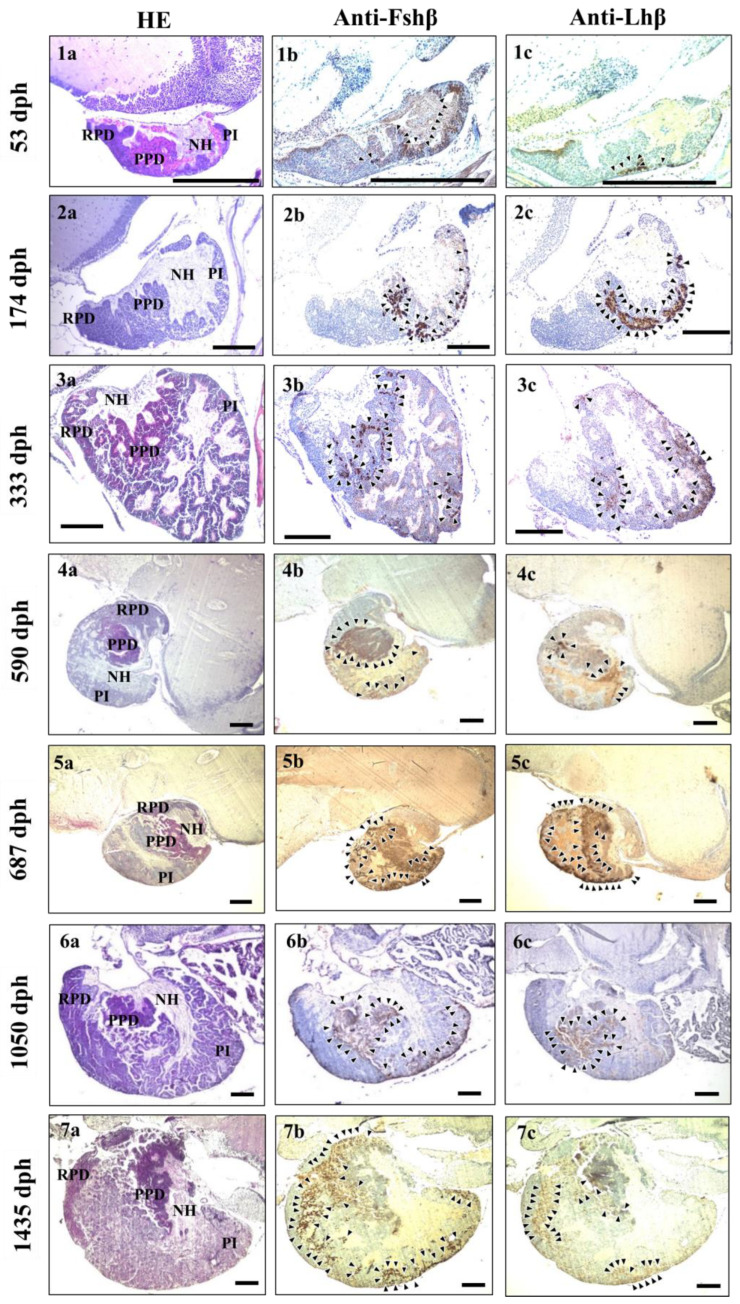
Pituitary structures and immunoreactive signals of Fshβ- and Lhβ-producing cells from 53 to 1435 dph in the longtooth grouper, *Epinephelus bruneus*. (**1a**–**7a**) Pituitary structures stained using haematoxylin and eosin. (**1b**–**7b**) Anti-Fshβ signal detection. (**1c**–**7c**) Anti-Lhβ signal detection. Counterstained with haematoxylin in immunohistochemical assay. NH, neurohypophysis; RPD, rostral pars distalis; PPD, proximal pars distalis; PI, pars intermedia. Arrows indicate positive signals. Scale bar = 200 µm.

HE-stained pituitary gland sections are shown in Figure 3(1a–7a). The pituitary structures consisted of the neurohypophysis and adenohypophysis (AH) from the anterior to the posterior direction. The AH was divided into three minor lobes based on the different coloured stains and boundaries: the rostral pars distalis, proximal pars distalis (PPD), and pars intermedia (PI).

The sagittal sections showed good immunoreaction with anti-Fshβ and -Lhβ, and were used to visualise the appearance and distribution of Fshβ and Lhβ immune signals in the pituitary gland. Fshβ signals were detected in the PPD and PI areas at 53 and 1050 dph (Figure 3(1b,6b)), whereas Lhβ signals were detected only in the PPD area (Figure 3(1c,6c)). The Fshβ and Lhβ signals were detected in the PPD and PI areas at 174, 333, 590, 687, and 1435 dph (Figure 3(2b,c, 3b,c, 4b,c, 5b,c and 7b,c)). The signal intensity of Fshβ and Lhβ cells was determined by a brown staining shade. Fshβ and Lhβ signals appeared weak at 53 dph, whereas the intensity increased at 174 and 333 dph, and showed a sharp increase from 590 to 1435 dph.

### 3.3. Gene Expression of gnrh1 in the Brain and fshβ and lhβ in the Pituitary Gland

**Figure 4:**
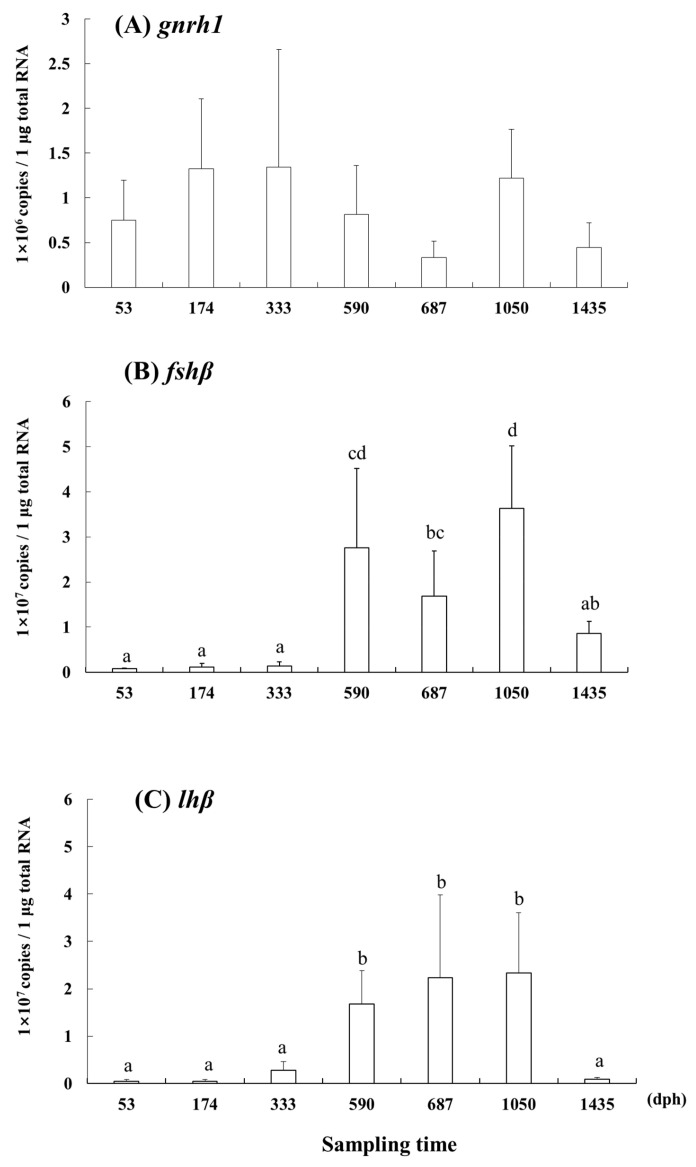
(**A**) *gnrh1.* (**B**) *fshβ.* (**C**) *lhβ*. Gene expression of *gnrh1* in the brain and *fshβ* and *lhβ* in the pituitary gland from 53 to 1435 dph in the longtooth grouper, *Epinephelus bruneus*. Data are shown as means ± SD. Data points with different letters (a–d) indicate significant differences according to Tukey’s HSD test (*p* < 0.05).

The expression of *gnrh1* was significantly higher at 174 and 1050 dph than at 687 and 1435 dph (*p* < 0.05; Figure 4). The expression of *fshβ* was significantly higher at 590, 687, and 1050 dph than at 53, 174, and 333 dph (*p* < 0.05). The expression of *lhβ* was significantly higher at 590, 687, and 1050 dph than at the other stages.

### 3.4. E_2_ and T Concentrations in Serum

**Figure 5:**
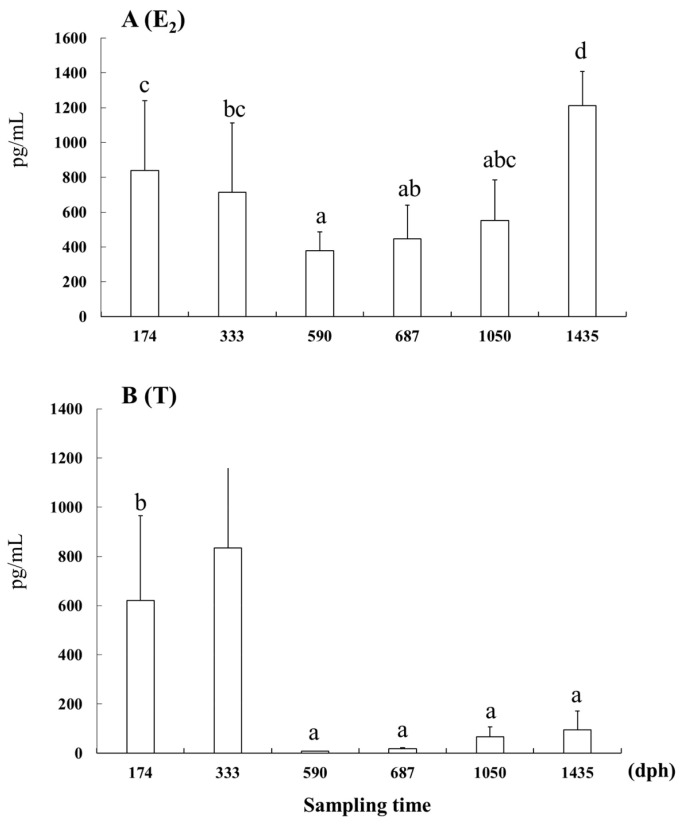
(**A**) E_2._ (**B**) T. Serum E_2_ and T concentrations from 174 to 1435 dph in the longtooth grouper, *Epinephelus bruneus*. Data are shown as means ± SD. Data points not sharing a letter (a–d) indicate a significant difference according to Tukey’s HSD test (*p* < 0.05).

The E_2_ concentrations were significantly higher at 174 and 333 dph than at 590 dph (*p* < 0.05) and were significantly higher at 1435 dph than those at any other stage (*p* < 0.05; Figure 5). The T concentrations at 174 and 333 dph were significantly higher than those at any other stage (*p* < 0.05).

## 4. Discussion

In the present study, we examined the gonadal development of the longtooth grouper and observed distinct phases of development. At 53 dph, gonadal development was in the undifferentiated phase. Subsequently, at 174 and 333 dph, the oocytes were in the oogonial phase, and the primary oocyte phase occurred between 590 and 1435 dph. The oogonial and primary oocyte phases observed in this study were longer than those previously reported. For example, Sao et al. (2012) [21] documented an undifferentiated phase ranging from 10 to 50 dph in longtooth groupers reared on Jeju Island, with the oogonial and primary oocyte phases extending from 140 to 210 dph and 270 to 365 dph, respectively. Additionally, Izumida et al. (2015) [2] reported the onset of maturation in longtooth groupers at approximately 4 years of age, while our specimens remained in the immature phase at 1435 dph. These variations in the timing of the oogonial and mature phases between studies could be attributed to habitat or rearing environment differences, such as variations in water temperature. Previous studies have shown that water temperature can directly influence maturation and spawning in various fish species, including the spotted snakehead, *Channa punctata* [24], and Caspian roach, *Rutilus rutilus caspicus* [25]. Consequently, the role of water temperature in gonadal development should be further explored in relation to these differences.

Devlin and Nagahama (2002) [26] reported strong correlations between steroid production and early gonadal development along the BPG axis. In this study, gonadal development at 53 and 174 dph occurred during the undifferentiated and ovarian phases, respectively. This suggests that ovarian differentiation likely took place between 53 and 174 dph in longtooth groupers. Previous studies have found that ovarian differentiation occurs between 47 and 140 dph in the Malabar grouper, *E. malabaricus* [27], and between 80 and 120 dph in the red-spotted grouper, *E. akaara* [19]. Additionally, these studies have shown that E_2_ levels were higher at the onset of ovarian differentiation and decreased towards the end of differentiation in both species [19,28]. In this study, the E_2_ level at 53 dph could not be detected due to challenges in obtaining blood samples from small specimens. Therefore, the specific E_2_ changes during ovarian differentiation in longtooth groupers remain unclear.

Our study revealed significantly higher E_2_ and T levels at 174 and 333 dph, respectively, during the oogonial proliferation phase. Androgens can be converted to oestrogens by aromatase, serving as both androgen and oestrogen precursors [29]. Moreover, research has shown that T may function as a precursor for E_2_ synthesis [30,31]. In the catfish, *Heteropneustes fossilis*, T present in the plasma vitellogenin serves as a precursor for oestrogen synthesis during ovarian recrudescence [32]. In addition, Wingfield and Grimm (1977) [33] observed an inverse relationship between plasma T and E_2_ levels during the annual reproductive cycle of *Pleuronectes platessa*. While studies have predominantly explored the role of T as a precursor or inhibitor of E_2_ during the maturation phase in teleosts, limited information is available regarding the oogonial proliferation phase for comparison with our findings. The observed increase in T levels during oogonial proliferation in our study may be due to its precursor, triggering E_2_ synthesis. For example, Yaron and Levavi-Sivan (2011) [34] reported that oogonia proliferated mitotically under E_2_ stimulation in teleosts, demonstrating that E_2_ directly influenced oogonial proliferation in the Japanese huchen, *Hucho perryi* [35]. Thus, the elevated E_2_ levels observed in our study may be associated with oogonial proliferation in longtooth groupers.

During the primary oocyte development phase from 590 to 1435 dph, our study detected a significant initial decrease in E_2_ levels at 590 dph, followed by increases at 687 and 1050 dph and a final significant increase at 1435 dph. Previous studies have reported low E_2_ levels during pre-vitellogenesis, followed by a significant increase during vitellogenesis in various fish species, such as the Arctic charr, *Salvelinus alpinus* [36]; the Russian sturgeon, *Acipenser gueldenstaedtii*; and the stellate sturgeon, *A. stellatus* [37]. Additionally, E_2_ levels were insufficient to trigger vitellogenesis in captivity in the dusky grouper, *E. marginatus* [38]. These findings suggest that substantial increases in E_2_ levels occur only when vitellogenin production necessitates significant E_2_ stimulation to initiate vitellogenesis in most teleosts. Based on previous reports, vitellogenesis in captively reared longtooth groupers occurs between April and May [39] and at approximately 4 years of age [2]. In this study, the increased E_2_ level at 1435 dph suggests its potential association with pre-vitellogenesis and vitellogenesis preparation.

Furthermore, Fshβ and Lhβ immune signals were detected in the PPD and PI areas at most stages. Notably, at 53 and 1050 dph, Fshβ signals were detected exclusively in the PPD and PI areas, whereas Lhβ signals were detected only in the PPD area. These results are consistent with those of other studies that have previously discussed the different distribution patterns of Fshβ and Lhβ cells in the PPD and PI areas (type-1) or the PPD area (type-2) [19]. Additionally, weak immune signals of Fshβ and Lhβ were observed at 53 dph before ovarian differentiation, whereas the signal intensity increased at 174 dph during oogonial proliferation. Similar results have been reported for red-spotted groupers [19]. Furthermore, *fshβ* and *lhβ* gene expression has been identified in the differentiating gonad of rainbow trout, *Oncorhynchus mykiss* [40]. However, Fshβ and Lhβ protein cells appeared right before gonadal differentiation in the pejerrey. In this study, both the immune signals and gene expression of Fshβ and Lhβ were detected at 174 and 333 dph during the oogonial proliferation phase. However, previous studies have indicated that only Fshβ signals were detected during the oogonial phase in the mummichog [41] and Japanese medaka, *Oryzias latipes* [42].

The signal intensities of Fshβ and Lhβ increased greatly at 590, 687, and 1050 dph during primary oocyte development, and *fshβ* and *lhβ* gene expressions were significantly higher during these stages. Consistent with our findings, Fshβ and Lhβ immune signals have been detected during primary oocyte development in the Malabar grouper [43], and Fshβ and Lhβ signal intensity increased greatly during the same phase in the red-spotted grouper [20]. However, the Fshβ signal was detected in the primary growth oocyte, and the Lhβ signal was found only in the secondary growth oocyte in the gilthead seabream [10]. Furthermore, Fshβ signals were only detected during primary oocyte growth in the mummichog [41] and chub mackerel, *Scomber japonicus* [44]. In the ricefield eel, *Monopterus albus*, Lhβ signals were first detected in the early primary growth oocytes; however, Fshβ signals were not detected until the secondary growth oocyte [11]. In the present study, stronger immune signals and higher gene expression of Fshβ and Lhβ simultaneously appeared, suggesting that both hormones may be active during primary oocyte development in the longtooth grouper.

## 5. Conclusions

Based on our findings, we can draw the following conclusions: (1) Fshβ and Lhβ signals were detected even before ovarian differentiation, with their intensity increasing notably during oogonial proliferation; (2) Fshβ and Lhβ exhibited more robust signals and higher transcription levels from 590 to 1050 dph than those observed at earlier stages, indicating that both hormones were active during primary oocyte development; (3) significant increases in E_2_ concentrations were observed at 174, 333, and 1435 dph, while T concentrations showed significant increments at 174 and 333 dph. These findings suggest potential correlations between E_2_ concentrations in the serum and the phases of oogonial proliferation and pre-vitellogenesis.

This study has shed light on the roles of Fshβ, Lhβ, and E_2_ in early gonadal development in the longtooth grouper. Artificial propagation and seedling production techniques have not yet been fully successful in this species. To gain a comprehensive understanding of the endocrine regulation mechanism throughout the reproductive cycle, further research should focus on monitoring the changes in these reproductive hormones within the BPG axis during maturation and sex change phases in the longtooth grouper.

## Figures and Tables

**Table 1:** Number of fish, total length (TL), body weight (BW), gonad weight (GW), and gonadosomatic index (GSI) for each age of the longtooth grouper, *Epinephelus bruneus*.

Sampling Date	dph	*n*	TL (cm)	BW (g)	GW (g)	GSI (%)
7 August 2015	53	20	3.69 ± 0.14	0.53 ± 0.04	UD	UD
7 December 2015	174	15	15.74 ± 1.21	58.73 ± 3.40	UD	UD
13 May 2016	333	15	18.11 ± 1.25	92.59 ± 11.54	UD	UD
2 February 2017	590	15	25.29 ± 2.57	219.20 ± 20.65	0.06 ± 0.02	0.03 ± 0.01
9 May 2017	687	15	26.96 ± 1.31	257.50 ± 12.56	1.12 ± 0.02	0.05 ± 0.02
12 May 2018	1050	15	31.87 ± 2.63	448.44 ± 30.61	0.74 ± 0.18	0.16 ± 0.04
30 May 2019	1435	15	39.98 ± 2.76	899.90 ± 56.64	1.59 ± 061	0.18 ± 0.04

dph, days post-hatching. *n*, number of sampled fish. UD, undetected.

**Table 2:** Sequences of primers used for real-time PCR.

Gene	Accession No.		Sequence (5′ → 3′)	Amplicon (bp)	Efficiency
*gnrh1*	FJ380047	Forward	CCACTGTCAGCTCTGGTCAT	60	1.998
		Reverse	AGGCTGTCCAGATCCCTCTT		
*fshβ*	EF583919	Forward	CTGCCACTCCGACTGTCATC	101	2.044
		Probe	ACCAGCATCAGCATCCCTGTGGAGA		
		Reverse	GGTAACACTGTCCTTCACATATGG		
*lhβ*	EF583920	Forward	TTTGAGCTTCCTGACTGTCCTC	115	2.015
		Probe	ACCCGACTGTCACCTACCCTGTGGC		
		Reverse	GGCTCTCGAAGGTGCAGTC		

## Data Availability

Data will be made available on request.

## Supplementary Materials

The following supporting information can be downloaded at: https://www.mdpi.com/article/10.3390/cells12222634/s1, Figure S1: Serially diluted samples containing cDNA derived from 50 ng, 5 ng, 500 pg, 50 pg, and 5 pg of total mouse RNA as starting template were amplified using the Fast-Start Essential DNA Probes Master. As negative control, temp late cDNA was replaced by PCR grade water. Figure S2: Detection of amplification products and plasmids on agarose gel.
