# Supplementary material for: Chronological Changes in Gonadotropin-Releasing Hormone 1, Gonadotropins, and Sex Steroid Hormones along the Brain–Pituitary–Gonadal Axis during Gonadal Sex Differentiation and Development in the Longtooth Grouper, Epinephelus bruneus
Source: Cells. 2023 Nov 16;12(22):2634. doi: 10.3390/cells12222634 (PMC10670822; doi:10.3390/cells12222634)
Supplement: Supplementary file 1 [file cells-12-02634-s001.zip › cells-2629522-supplementary.pdf]

For life science research only.
Not for use in diagnostic procedures.

Roche

Print Protocol | Print Changes to Previous Version | Print License Disclaimer

FastStart Essential DNA Probes Master

Version 04

Content version: January 2013

Ready-to-use hot start reaction mix for real-time PCR with the LightCycler® Nano System and LightCycler® 96 System

**Cat. No. 06 402 682 001**	5 × 1 ml (5 × 100 reactions, 20 µl each)
**Cat. No. 06 924 492 001**	10 × 5 ml (10 × 500 reactions, 20 µl each)

Store the kit at −15 to −25°C

The optimal probe concentration is the lowest concentration that results in the lowest Cq and an adequate fluorescence for a given target concentration.

⚠ For a digestible hybridization complex to form correctly, the hydrolysis probe must anneal to the target before primer extension. The T_m of the probe should be only slightly higher than the T_m of the PCR primer, so the hybridization complex is stable. Furthermore, the probe sequence must account for mismatches in the DNA template, since these will also affect the annealing temperature.

$MgCl_2$

The reaction mix in this kit already contains an optimal concentration of $MgCl_2$, which works with nearly all primer combinations.

You do not need to adjust the $MgCl_2$ concentration to amplify different sequences.

2.2 Experimental Protocol

Program the LightCycler® Nano or LightCycler® 96 Instrument before preparing the reaction mixes.

A LightCycler® Nano or LightCycler® 96 Instument protocol that uses FastStart Essential DNA Probes Master should contain the following programs:

- **Pre-Incubation (Hold)** for activation of FastStart Taq DNA polymerase and denaturation of the DNA.
- **Amplification** of the target DNA.

For details on how to program the experimental protocol, see the LightCycler® Nano or LightCycler® 96 System Guides.

The following tables shows the PCR parameters that must be programmed for a LightCycler® Nano or LightCycler® 96 System PCR run with the FastStart Essential DNA Probes Master using LightCycler® 8-Tube Strips or LightCycler® 480 Multiwell Plates. The hold times shown here are a robust protocol (standard protocol) and can be shortened depending on your assay design[1].

Experimental Protocol

2.2.1 LightCycler® Nano Instrument Protocol

The following procedure is optimized for use with the LightCycler® Nano Instrument.

**Setup**			
**Run Settings**			
**Optics Settings**			
Hydrolysis Probes		Normal Quality	
**Profile**			
**Programs**			
Temp. (°C)	Ramp (°C/s)	Hold (s)	Acquire
**Hold**			
95	4	600 [3)]	
**2-Step Amplification**			
No. of Cycles: 45			
95	5	20 [1) 4)]	
60 primer dependent [2)]	4	40 [1) 4)]	✓

2.2.2 LightCycler® 96 Instrument Protocol

The following procedure is optimized for use with the LightCycler® 96 Instrument.

**Run Editor**			
**Detection Format**			
Dyes 1:		FAM	
**Programs**			
Temp. (°C)	Ramp (°C/s)	Hold (s)	Acquisition Mode
**Pre-incubation**			
95	4.4	600 [3)]	
**2-Step Amplification**			
No. of Cycles: 45			
95	4.4	10 [1) 4)]	
60 primer dependent [2)]	2.2	30 [1) 4)]	Single

1) For well-established assays (with amplicon size not exceeding 200 bp in length), you may shorten the amplification times to: 95°C for 10 seconds, and 60°C for 10-30 sec-onds. Fourty-five cycles are suitable for most assays. If the assay is optimized and has steep amplification curves and early crossing points (even when target con-centrations are low), 40 cycles should be sufficient. Reducing the number of cycles will reduce the time required for the assay (fast protocol).
2) For initial experiments, set the target temperature (the primer annealing tempera-ture) 5°C below the calculated primer T_m.
3) For some assays, a pre-incubation of 300 seconds is sufficient (fast protocol). How-ever, if high polymerase activity is required in early cycles, a 600-second period is recommended, especially for higher reaction volumes and when working with unpu-rified cDNA samples as template. Do not use more than 2 µl unpurified cDNA sam-ple.
4) For greater precision in target quantification experiments, it can be advantageous (in some cases) to choose longer annealing and extension times for the amplification cycles. This is especially recommended for higher reaction volumes.

Preparation of the PCR Mix

Follow the procedure below to prepare one 20 µl standard reaction.

⚠ Always wear gloves during handling.

❶
- Thaw one vial of "FastStart Essential DNA Probes Master" (Vial 1, red cap) and Water, PCR grade (Vial 2, colorless cap).
- Briefly spin vials in a microcentrifuge before opening to ensure recovery of all the contents.
- Mix carefully by pipetting up and down and store on ice.

❷ Prepare a 10× concentrated solution that contains PCR primers and hydrolysis probe.

❸ In a 1.5 ml reaction tube on ice, prepare the PCR mix for one 20 µl reaction by adding the following components in the order listed below:

Component	Volume
Water, PCR grade (Vial 2, colorless cap)	3 µl
Primer-probe mix [1], 10× conc.	2 µl
Master Mix, 2× conc. (Vial 1, red cap)	10 µl
**Total Volume**	**15 µl**

1) Due to possible primer/primer interactions that occur during storage, it may be necessary to preheat the PCR primer-probe mix for 1 minute at 95°C before starting the reaction. This extra step will ensure optimum sen-sitivity.

⚠ To prepare the PCR mix for more than one reaction, multiply the amount in the "Volume" column above by z, where z = the number of reactions to be run + sufficient additional reactions.

3. Results

Quantification Analysis

The following amplification curve was obtained by using the FastStart Essential DNA Probes Master on the LightCycler® Nano Instrument in combination with the β-Actin RealTime ready Assay No. 1007903, targeting mouse β-Actin mRNA. The intensity in relative fluorescence units (RFU) versus cycle number is displayed (see Figure 1).

2-step protocol (95°C 10 minutes; 45 × 95°C 10 seconds, 60°C 10 seconds)

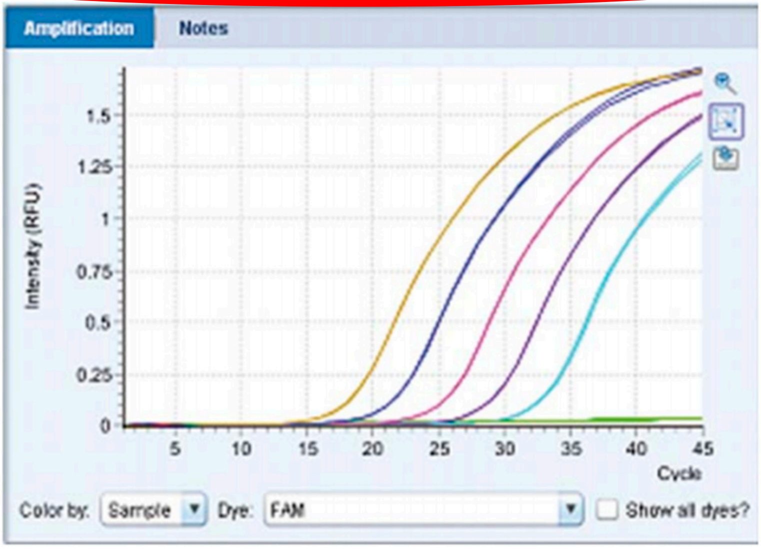

Figure S1: Serially diluted samples containing cDNA derived from 50 ng, 5 ng, 500 pg, 50 pg, and 5 pg of total mouse RNA as starting template were amplified using the FastStart Essential DNA Probes Master. As negative control, template cDNA was replaced by PCR grade water.

Amplification Curves

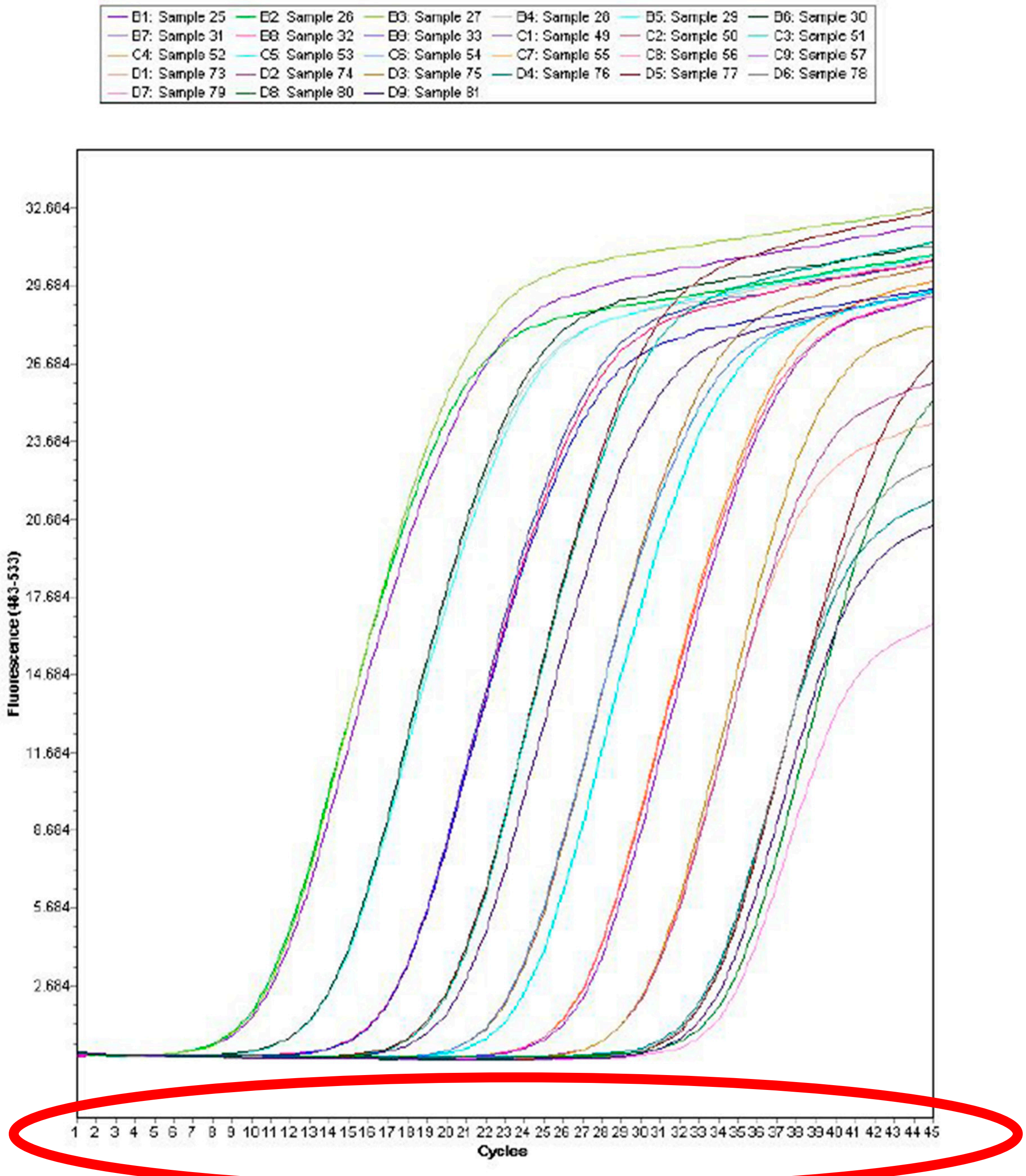

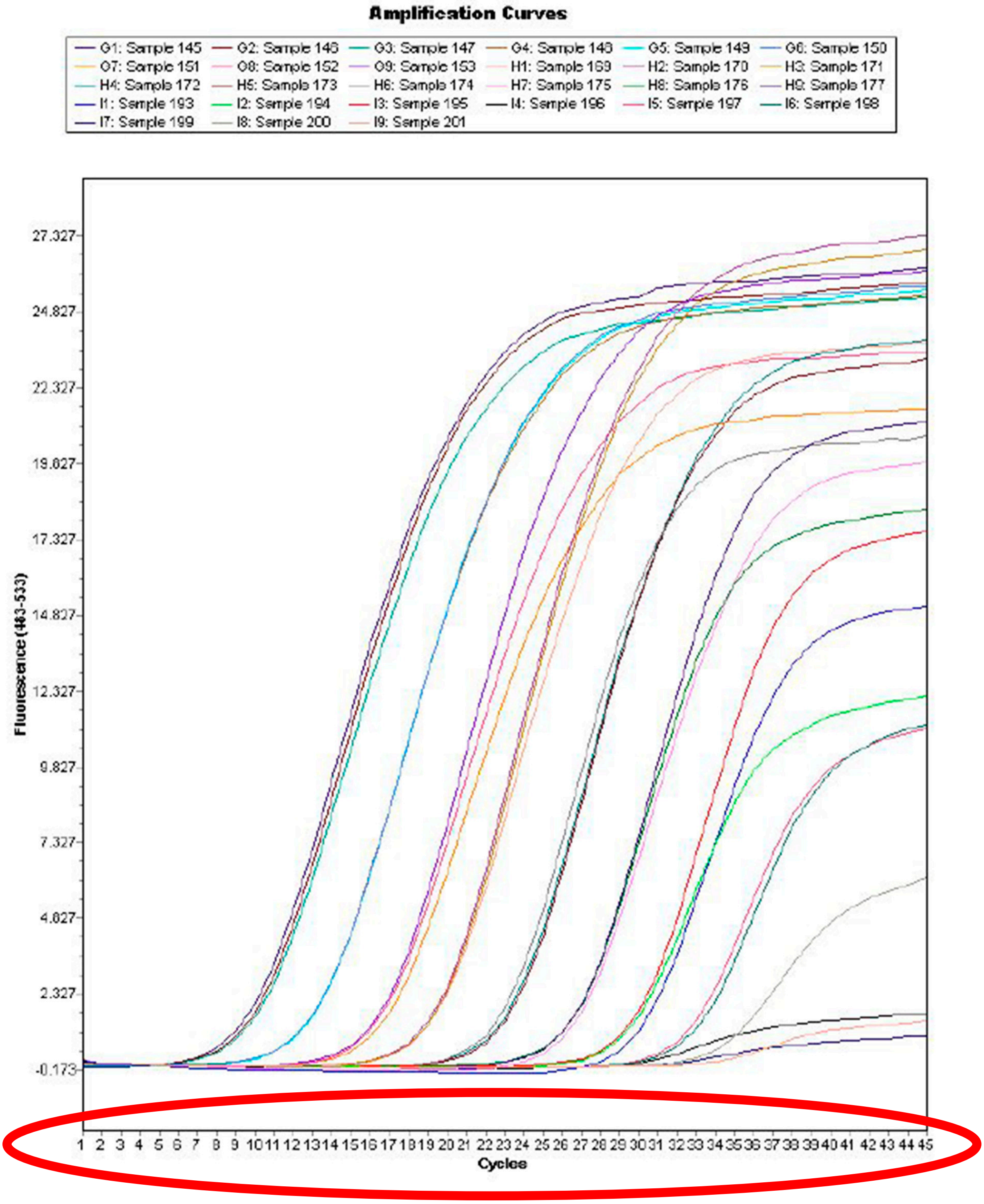
Amplification Curves
G1: Sample 145
G2: Sample 146
G3: Sample 147
G4: Sample 148
G5: Sample 149
G6: Sample 150
G7: Sample 151
G8: Sample 152
G9: Sample 153
H1: Sample 169
H2: Sample 170
H3: Sample 171
H4: Sample 172
H5: Sample 173
H6: Sample 174
H7: Sample 175
H8: Sample 176
H9: Sample 177
I1: Sample 193
I2: Sample 194
I3: Sample 195
I4: Sample 196
I5: Sample 197
I6: Sample 198
I7: Sample 199
I8: Sample 200
I9: Sample 201
Fluorescence (483-533)
Cycles

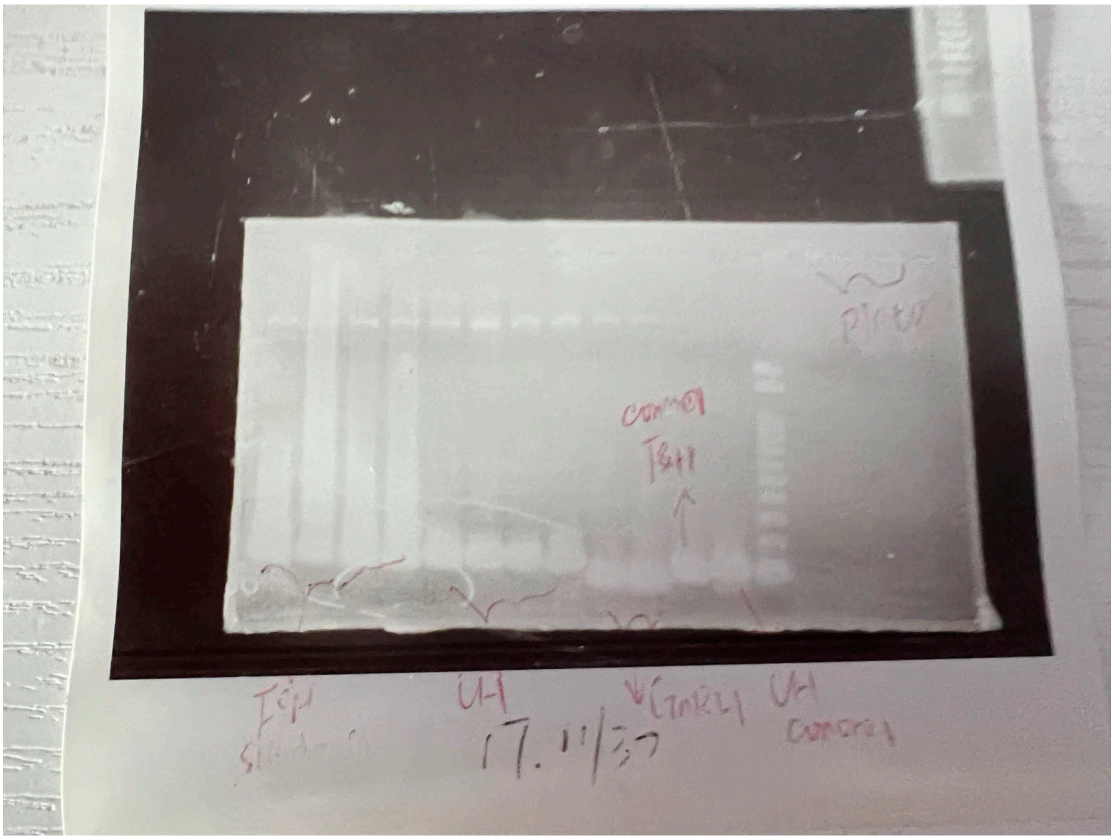
UH
GnRH
UH
17.11/30

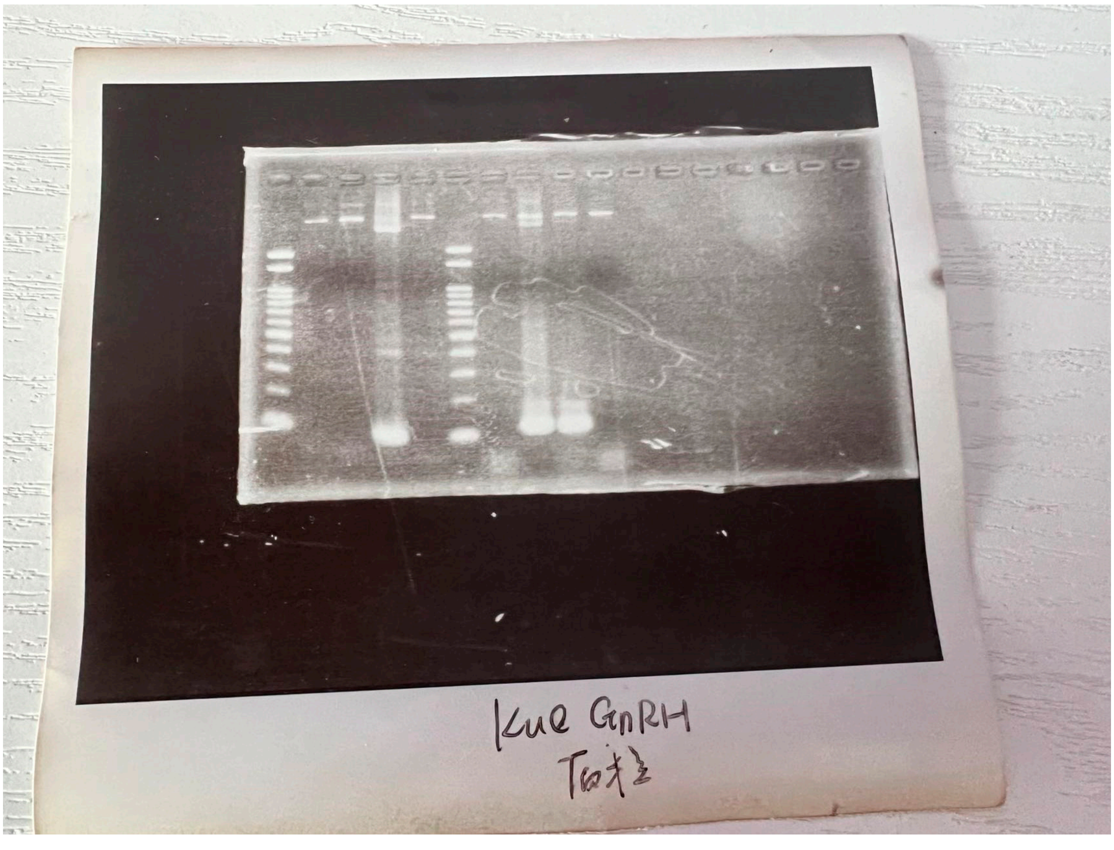
Kue GnRH

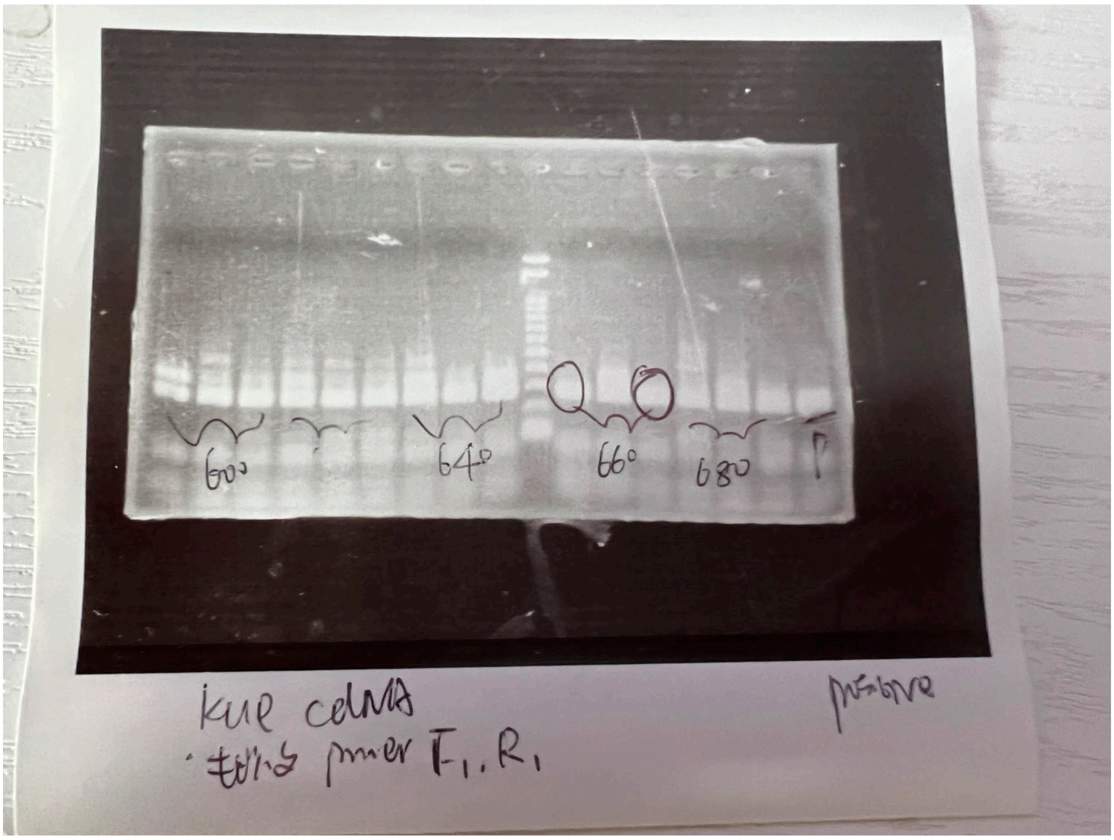
60°
64°
66°
68°
P
positive
primer F1, R1

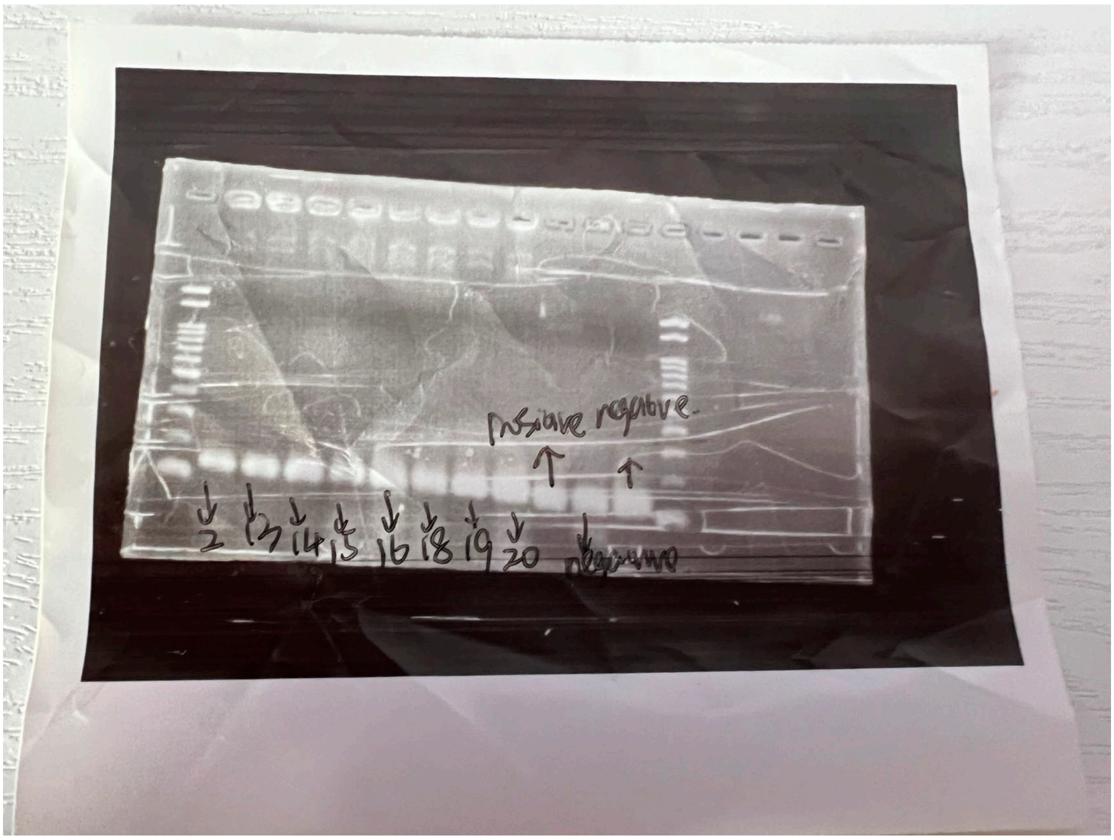
positive negative
12 13 14 15 16 18 19 20
negative

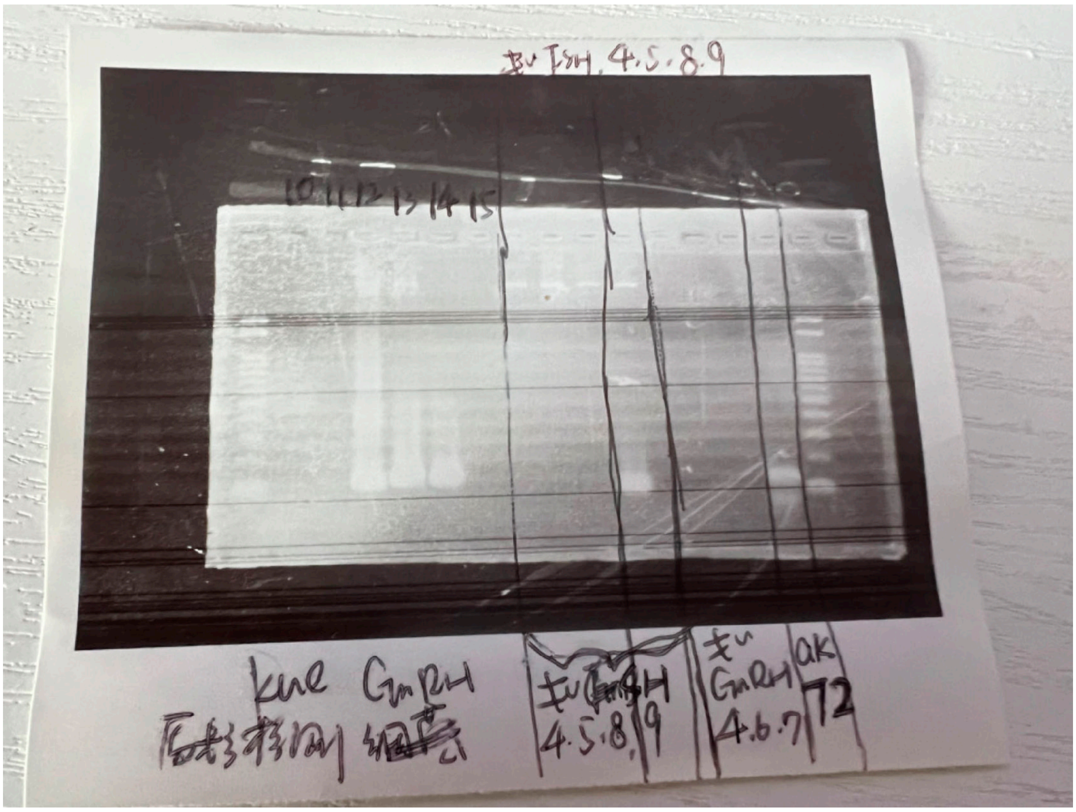

Figure S2: Detection of amplification products and plasmids on agarose gel
